# SiNiSan Ameliorates the Depression-Like Behavior of Rats That Experienced Maternal Separation Through 5-HT1A Receptor/CREB/BDNF Pathway

**DOI:** 10.3389/fpsyt.2019.00160

**Published:** 2019-03-28

**Authors:** Kerun Cao, Chongkun Shen, Yumei Yuan, Shasha Bai, Lei Yang, Lili Guo, Rong Zhang, Yafei Shi

**Affiliations:** ^1^School of Fundamental Medical Science, Guangzhou University of Chinese Medicine, Guangzhou, China; ^2^Shenzhen Baoan Hospital of Chinese Medicine, Shenzhen, China; ^3^Institute of Clinical Pharmacology, Guangzhou University of Chinese Medicine, Guangzhou, China; ^4^Third Affiliated Hospital of Henan University of Chinese Medicine, Henan University of Chinese Medicine, Zhengzhou, China

**Keywords:** SiNiSan, early life stress, depression, brain-derived neurotrophic factor, serotonin 1A receptor

## Abstract

**Background:** Early adverse life stress is an important dangerous factor in the development of psychiatric disorders, particularly depression. Available clinical antidepressant agents, such as fluoxetine, [a selective serotonin reuptake inhibitor (SSRI)], are unsatisfactory because of their side effects. SiNiSan (SNS) is a classic Chinese medicine prescription regarded to disperse stagnated liver qi to relieve qi stagnation. Therefore, this study was designed to detect the effects and molecular mechanism of SNS treatment in rats subjected to maternal separation (MS).

**Method:** Male neonatal Wistar rats were divided into six groups including control + ddH_2_O, MS + ddH_2_O, MS + fluoxetine (5 g/kg), MS + SNS -low dose (2.5 g/kg), MS + SNS -medium dose (5 g/kg), MS + SNS -high dose (10 g/kg). The volume of drugs and ddH2O in each group are according to the weight of rats every day (10 mL/kg). Each group comprised 16 pups with 8 young and 8 adult pups. Except for the control group, all MS groups were separated from their mothers for 4 h/day from 9:00 to 13:00 during postnatal days (PNDs) 1 to 21. After MS, the six groups were intragastrically administered with ddH2O, fluoxetine, and different doses of SNS until PND 28 (for young pups) and PND 56 (for adult pups). The pups were weighed every day, and depression-like behavior was assessed by sucrose preference test, open field test, and forced swimming test. Serotonin 1A (5-HT1A) receptor, phosphorylated protein kinase A (p-PKA) substrate, cAMP response element-binding protein (CREB), p-CREB and brain-derived neurotrophic factor (BDNF) in the hippocampus were examined by Western blot, and *in situ* 5-HT1A receptor expression was measured by IHC.

**Results:** Young and adult MS rats exhibited depression-like behavior. However, the depression-like behavior was ameliorated by SNS in both age groups. The levels of 5-HT1A receptor, p-CREB, and BDNF in the hippocampus were reduced in young and adult MS rats. SNS treatment significantly up-regulated the expression of 5-HT1A receptor, p-CREB, and BDNF in the hippocampus of adult MS rats. However, few significant effects on the protein expression were observed in the young MS rats.

**Conclusion:** MS in infancy could develop depression-like behavior in young and adult. SNS treatment may perform antidepressant effects on young and adult MS rats through the BDNF/PKA/CREB pathway.

## Introduction

Adverse stress in early life is an important dangerous factor for suffering from many types of mental disease, such as depression, posttraumatic stress disorder, and schizophrenia. Several reports have shown that adversity in early life is closely related to the occurrence and development of depression, which is likely to be accompanied with impulsive, suicidal, and self-injurious behavior (1, 2). Maternal separation (MS) in rats, which temporarily deprives pups from their mothers to a new environment during lactation, has been established as a model to replicate early life adversity in animal experiments (3). MS has been suggested to induce depression-like behavior in rodents, such as behavioral despair during forced swimming test (FST) and anhedonia in sugar water preference test (4, 5). In addition, MS could trigger anxiety-like behavior in the open field test (OFT) (6). On one hand, for juvenile and adult rats, postnatal MS procedure could downregulate new born cells in the hippocampus and granule cells in the dentate gyrus of the hippocampus. Thus, MS may affect the development and neuroplasticity in the hippocampus of rats (7). These results indicate that the emotions and behavior of individuals could be significantly affected by postnatal MS in the long term.

The change in the serotonin system has been demonstrated to be closely related with mental disorders, but this relationship needs further investigation (8, 9). Serotonin 1A (5-HT1A) receptor is closely related to emotional disorders. Moreover, the brain-derived neurotrophic factor (BDNF) has been increasingly investigated for depression in animal models. BDNF is generally distributed in the hippocampus and participates in neurogenesis and development of the hippocampus. Previous study showed that the expression of BDNF in the hippocampus of rats that experienced postnatal MS declined with age but increased in the medial prefrontal cortex for normal rats. BDNF significantly decreased in these two regions during the early adulthood of rats (10). A systematic review of the change in BDNF expression using the social isolation model of rats revealed that BDFN could be downregulated in the hippocampus by stress during postnatal period but not altered in the cerebral cortex (11). Another study has reported the correlation of serotonin and BDNF and showed that these molecules played to some extent crucial roles in neuroplasticity and were associated with certain neurological diseases, especially during aging (12). Furthermore, the relative signaling pathways of BDNF caused by early life stress have been studied to explore the mechanism of depression and antidepressants. BDNF and CREB/BDNF signaling pathways, including the expression of BDNF, phospho-cAMP response element-binding protein (CREB) (pCREB), phospho-ERK1/2, phospho-Protein kinase B (AKT), and TrkB (a receptor of BDNF), were downregulated in the cerebral hippocampus of depression models that underwent stress (13, 14). Moreover, numerous studies have shown certain correlation between CREB and the pathogenesis of depression as well as the mechanism of antidepressants. CREB, which is regarded as a crucial transcription factor, could be activated by various signaling pathways. Protein kinase A (PKA), which is the upstream signal for CREB, may be associated with the pathogenesis of depression. Several studies have reported that the disorder of adenosine cyclophosphate (cAMP)-PKA-CREB-BDNF signaling pathway in the hippocampus of animals could be involved in the pathophysiological process of depression (15, 16).

Monoamine-based antidepressants, such as selective serotonin reuptake inhibitors (SSRIs), are frequently used for depression. However, SSRIs, such as fluoxetine, are unsatisfactory for depression because of their side effects, such as delayed onset of ~6–8 weeks after taking the medicine and the insensitivity for the drug by some patients (17, 18). In traditional Chinese medicine, the primary pathogenesis of depression is emotional upset and stagnation of qi in the liver. Depression belongs to melancholia in traditional Chinese medicine, and the treatment should be based on soothing the liver and relieving depression. SiNiSan comes from the Treatise on Febrile Diseases, and is mainly used to regulate the liver-qi. This compound is considered the most fundamental formula on soothing the liver and resolving depression. Moreover, several animal experiments have demonstrated the effects of SiNiSan on depressive behavior, such as recovering the loss of weight, anhedonia in sucrose preference test (SPT), and low activity in OFT for CUMS models (19). However, few studies have focused on the effect of SiNiSan treatment for depression triggered by early life adversity through the signaling pathway of 5-HT1A receptor/CREB/BDNF. Although these findings have indicated the depression-like behavior caused by postnatal MS and the effect of SiNiSan on depression, the effect of SiNiSan on depression induced by postnatal MS in young and adult rats is rarely investigated.

In the present study, we explored the effect of SiNiSan treatment on young and adult Wistar rats maternally separated during early development by behavioral tests and expression detection of several relative proteins in the 5-HT1A receptor/CREB/BDNF signaling pathway. The results provided valid evidence for the clinical use of SiNiSan on depressive patients who experienced MS during childhood.

## Materials and Methods

### Animals

The 20 Wistar pregnant rats with 16-day gestation were bought from the Experimental Animal Center in Guangzhou University of Chinese Medicine and housed in a controlled environment. All animals were raised on a 12 h light/12 h dark cycle (lights on at 19:00) and with free access to food and water. The environmental conditions were maintained at 22°C and relative humidity of 40–70%. All pregnant rats produced ~8–10 pups per dam, and 91 male pups were selected from all 20 dams for the experiment. At the end of molding at postnatal day (PND) 21, the male pups were randomly divided into six groups based on the weight, namely, control group (non-MS), model group (MS administration), positive group (fluoxetine treatment), low-dose SNS treatment (SNS-L), medium-dose SNS treatment (SNS-M), and high-dose SNS treatment (SNS-H). All experimental procedures strictly followed the guidelines of the International Association for the Use of Animals in Research and permitted by the Committee of Animal Experiment Ethics Review in Guangzhou University of Chinese Medicine.

### Maternal Separation

The day of birth was regarded as PND 0. The groups, except the control, were all maternally separated from PND 1 to PND 21, while the control group was undisturbed. This procedure was according to the method employed in a previous study (10). One male pup from each litter was randomly left in the cage with his mother, and marked as the control group (NMS group). The rest of the male pups were separated from their mothers to another room from PND 1 to PND 21 for 4 h per day (from 10:00 to 14:00). In addition, cotton was laid on the bottom plate of the pup cage to keep the pups warm from PND 1 to PND 10. During the separation, pups from the same litters were placed in the same pup cage but isolated from each other by several hardboards to avoid contact. After weaning at PND 21, the medicine group was administered with medicine intervention. All male rats underwent behavioral test during young stage (PND 22–28) and adult stage (PND 50–55) and sacrificed to gather complete brains and hippocampus for Western blot (WB) test and immunohistochemistry (IHC) at PND 28 and PND 56.

### Drug Treatment

**Figure 1:**
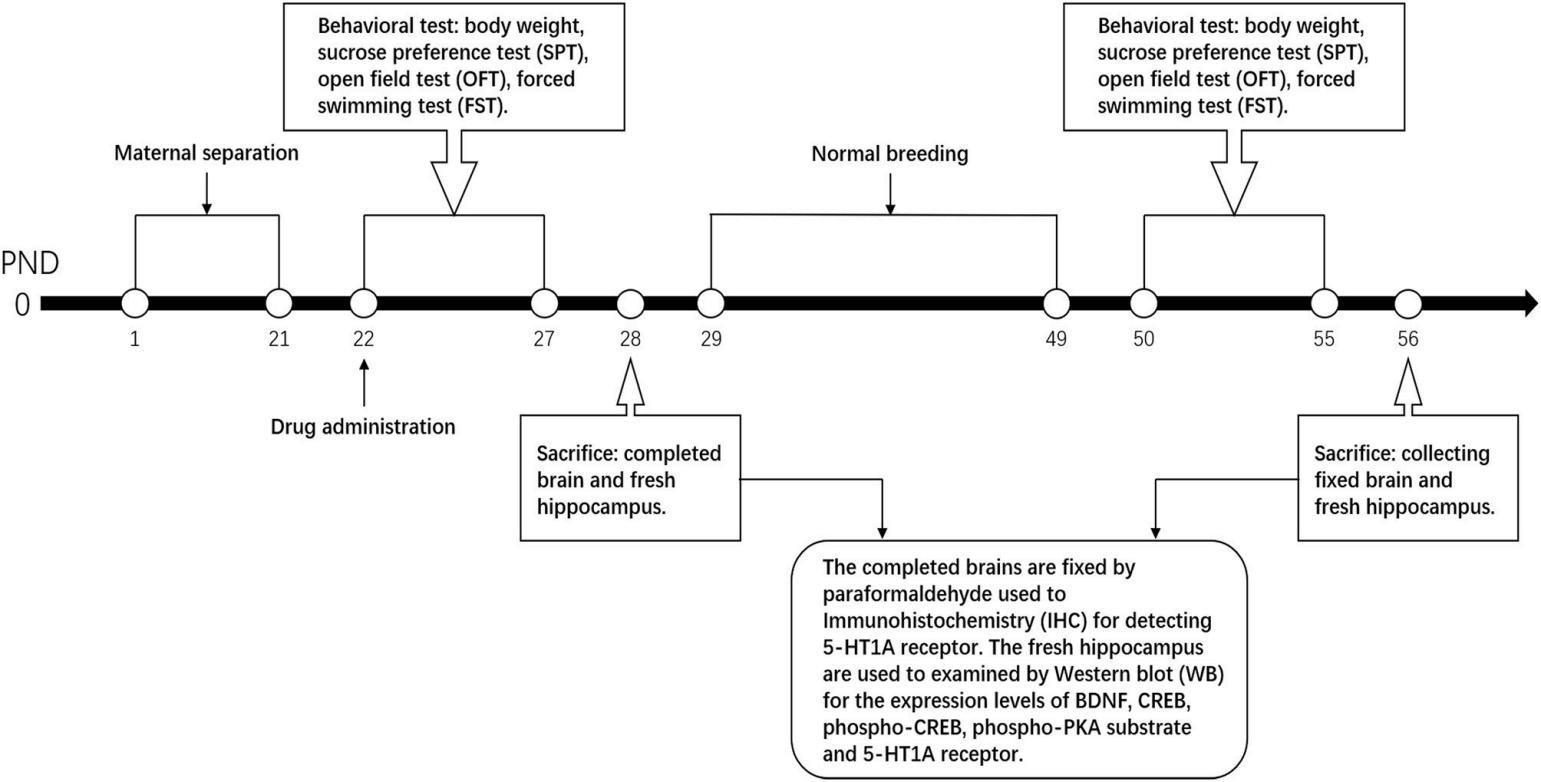
Process time points. The process time points are shown by the timeline.

SNS is composed of four Chinese medicinal herbs, namely, Zhigancao, Zhishi, Chaihu, and Shaoyao with equal ratio at 1:1:1:1. SNS was bought from the First Affiliated Hospital of Guangzhou University of Chinese Medicine. The herbs were ground into coarse powder and soaked in ten times volume of ddH_2_O more than the herb for 60 min. After boiling by strong fire, the herbs were heated by slow fire for 40 min. After cooling, the decoction was filtered using several layers of gauze to collect the colature concentrated to 1 g/mL of raw drug by a rotary evaporator. The Quality Control of Sinisan was performed by HPLC. It was performed on an Agilent Technologies 1260 Infinity system with a 1260 DAD VL detector at 240 nm. Kromasil Cl8 (250 mm × 4.6 mm, 5 μm) column was used and the temperature was set at 30°C. The mobile phase was consisted of acetonitrile (A) and water containing 0.1% phosphoric acid (B). A linear gradient elution program was used as follows: 0–10 min, 0%–10% A; 10–20 min, 20%–25% A; and 20–28 min, 25%–29% A, 28–40 min, 29%–40% A , 40–50 min, 40%–55% A, 50–60 min, 55%–73% A. The flow rate was set at 1.0 mL· min^−1^. The injection volume was 10 μL (Supplementary Materials). Considering low dose as an equivalent dose, the low dose was calculated from the equivalent dose coefficient conversion on people and rats. The low, medium, and high doses of SNS were 0.25, 0.5, and 1 g/mL, respectively. Fluoxetine was bought from the First Affiliated Hospital of Guangzhou University of Chinese Medicine and dissolved in ddH_2_O to 0.5 mg/mL, which also depended on the equivalent dose coefficient conversion on people and rats. After molding at PND 22, the groups were intragastrically intervened by ddH_2_O, fluoxetine, and different doses of SNS rats until PND 28 (young rats) and PND 56 (adult rats). The process time points are shown in Figure 1.

### Behavioral Test

#### Body Weight (BW)

**Figure 2:**
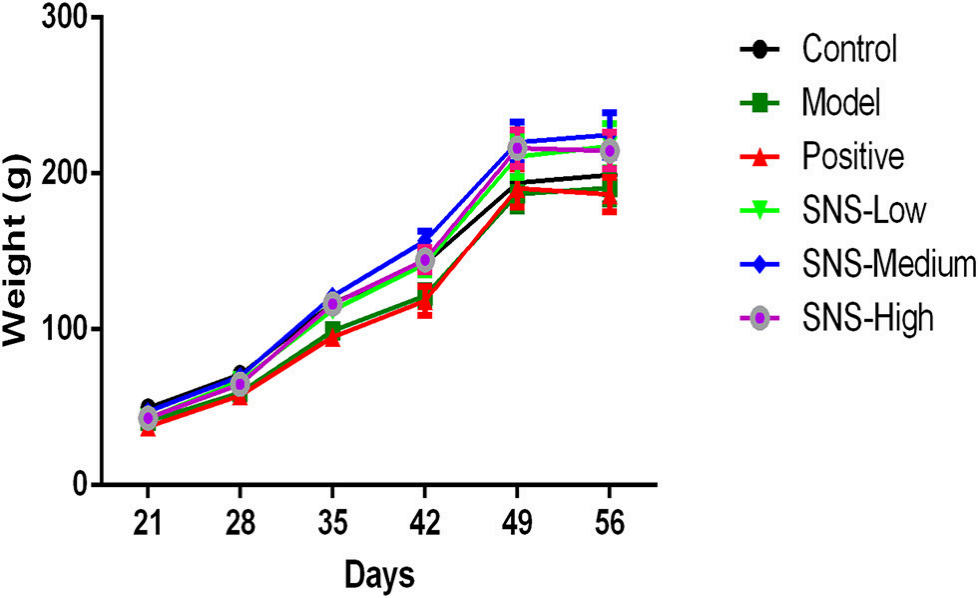
Effects of SNS on the changes in body weight (BW). The effect of SNS on the changes in the BWs of MS male rats during PND21–26 was expressed in the graph. The weights on PND 21, 35, 42, 49, and 56 are presented as means ± SEM.

The change in BW was compared with the baseline data measured at PND 21 to evaluate the impact of MS on the food preference. The data were recorded per day and measured on PNDs 21, 28, 35, 42, 49, and 56 to observe the weight variation and alteration in BW gain (Figure 2, Table 1).

**Table 1:** The effects of SNS on weight increase of MS male rats (g).

	**PND28**	**PND35**	**PND42**	**PND49**	**PND56**
Control	21.64 ± 1.00	66.73 ± 1.85^*^	92.43 ± 3.34	147.40 ± 3.09	152.31 ± 2.62
Model	18.24 ± 0.80	58.36 ± 2.77	80.72 ± 5.32	148.80 ± 8.97	152.86 ± 8.57
Positive	19.98 ± 1.13	57.82 ± 2.98	81.13 ± 7.36	149.88 ± 8.04	145.74 ± 6.90
SNS-low	23.98 ± 1.76	68.69 ± 2.81^*^	98.46 ± 6.67^*^	168.73 ± 11.21	175.69 ± 12.14
SNS-medium	22.51 ± 2.06	71.88 ± 2.11^*^	107.47 ± 6.23^**^	175.68 ± 9.74	180.53 ± 10.93
SNS-high	21.81 ± 1.30	70.62 ± 2.40^*^	99.03 ± 6.56^*^	172.36 ± 8.66	170.64 ± 8.61

*The body weights of all male rats were weighed per day from PND 21 after MS. The data of PND 21 were used as the starting point. Weight gain was measured on weeks 1, 2, 3, 4, and 5 at the age of PND 28, 35, 42, and 56. Differences were analyzed by one-way ANOVA with Fisher's LSD. Data are presented as means ±SEM*.

* *P < 0.05*,

** *P < 0.01 compared with the model group (statistically significant)*.

#### Sucrose Preference Test

The preference of sucrose intake by the male rats was evaluated by the decrease in the consumption of sucrose water. This behavior has been considered as an indicator of anhedonia, which usually appears in depression (5, 6). All rats were exercised to adapt to 1% sucrose solution during the period of adaptation. For the first 2 days, the rats were provided two bottles of 1% sucrose solution for 24 h, and then one bottle of water and one 1% sucrose solution for the second 24 h with an exchange of bottles at half time. Then, following a 24 h period of food and water deprivation, all rats placed in individual cages were tested simultaneously with two pre-weighed bottles, one bottle of sucrose (1% w/v) and the other water, for 2 h. The two bottles were exchanged one time at the middle time. After the test, the bottles of 1% sucrose solution and water were reweighted and recorded to calculate the liquid consumption. Sucrose preference was calculated from the following formula as applied in previous studies: Sucrose preference = {Sucrose consumption (g)/[Sucrose consumption (g) + water consumption (g)]} × 100. SPT was measured on PND 24 and PND 54 per rat (20). Anhedonia was presented by reduced sucrose preference.

#### Open Field Test

General curiosity and detective activity of each rat were detected to assess the anxiety-like behavior during OFT as described in previous studies (6). The open field apparatus consisted of a 100 × 100 cm square space with black walls and black baseplate (The Spontaneous Activity Open Field, Guangzhou Feidi Biotechnology Co. LTD). Each rat was carefully placed at the center of the open field floor and allowed to move independently and explore freely for 3 min. To evaluate the ability of the rats to adapt to a new environment and the detective activities, the time and distance spent in the central area were traced and recorded. These parameters are considered indices of locomotor activity. After each test, 75% ethyl alcohol was used to clean the urine and feces left behind by the other rats which could produce odor signals that would interfere the next rats to be tested. OFT was performed on PNDs 22 and 51.

#### Forced Swimming Test

The FST, especially for young rats, was originally described in a previous study (21). The rats were placed in a plexiglass cylinder with a diameter of 30 cm and a height of 50 cm. The cylinder was filled with ~30 cm of water at 23–26°C such that the rats could not touch the bottom of the cylinder. On the first day for exercise, the animals underwent a 15-min pre-test swim. After the pretest, the rats were immediately removed from the cylinder and mildly dried with a towel. After completely dried, the rats were returned to their home cages. On the second day after the pre-test, the rats were placed in a water-filled cylinder for the test, and the behavior was recorded by videotape for 5 min. The water in the cylinder was changed after each rat had been tested. After the test swim was completed, the rats were dried similarly as that during the pre-test. Then, the rats were returned to their cages until they were dried.

### Biochemical Assays

#### Western Blot Analysis

The rats in the six groups were sacrificed on PNDs 28 and 56. The animals were anesthetized with an injection of 10% chloral hydrate (0.35 mL/100 g, i.p.). Then, all rats were sacrificed by decapitation. Six rats per group were administered decollation to gather the fresh hippocampus from the brain. The hippocampus was quickly frozen in liquid nitrogen and then transferred to an −80°C refrigerator for the WB, which was performed as follows. The samples were homogenized with RIPA lysis buffer (strong) which was added with protease inhibitor cocktail for protein extraction at a proportion of 1:100. Then, the supernatant was collected after centrifugation at 12,000 rpm under 4°C for 15 min. The total protein content was determined using BCA assay. After the quantitative determination and addition of loading buffer for the total protein content, the proteins of each sample in the Eppendorf tube were denatured in water at 100°C for 5 min. Subsequently, protein samples were fractionated through 12% SDS-polyacrylamide gel electrophoresis. The proteins of samples were electro-transferred onto polyvinylidene difluoride membranes with constant current at 300 mA for 75 min. The membranes were blocked with 5% bull serum albumin-TBST for 3 h at room temperature. Protein expression was detected by incubation with rabbit polyclonal primary antibodies against BDNF (1:1,500), CREB (1:1,000), pCREB(1:1,000), pPKA substrate (1:1,000), 5-HT1A receptor (1:1,000), and rat polyclonal primary antibody GAPDH (1:5,000) at 4°C overnight. After incubation with the primary antibody, the membranes were incubated with goat anti-rabbit HRP-conjugated IgG (1:3000–5,000) and goat anti-rat HRP-conjugated IgG (1: 5,000) at room temperature for 1 h. All antibodies were dissolved in 0.5% blocking reagent. The bound antibodies were expressed using an enhanced chemiluminescence reagent by ECL kit (BIO-RAD Molecular Imager™ XRS+) and quantified using Image Lab™ software. The experiments were performed four times with four samples per group. In the WB analysis, GAPDH was used as loading control to normalize the levels of target protein detected. The mean optical density value of each protein band relative to that of the GAPDH band from the same sample was calculated.

#### Immunohistochemistry (IHC)

After sacrificing on PNDs 28 and 56, two rats from each group were administered with 4% paraformaldehyde solution perfusion to collect the complete brain samples. The samples were stored in the perfusion solution for IHC. Briefly, IHC was performed such that the fixed brain tissue with 4% paraformaldehyde solution was separated from the hippocampus, that is, 1.30–5.30 mm from the bregma. Then, the brain samples were dehydrated thrice for 30 min using different concentrations of ethyl alcohol (70, 90, 96, and 100%) and xylene and set in paraffin. After dewaxing by xylene twice for 8 min, ethanol (70, 80, 95, and 100%) for 5 min, and distilled water for 3 min, the sections were put into citrate buffer solution (0.01 M pH 6.0) and heated in a microwave oven. The slides were blocked using 6.5% BSA for 30 min at room temperature (RT) and incubated in a first rabbit anti-5-HT1A receptor polyclonal IgG (1:500) at 4°C overnight. After washing thrice in 0.05 M PBS for 5 min at RT, the sections were incubated with a secondary goat anti-rabbit IgG (1:1,000) for 1 h at RT and incubated in an avidin—biotin—peroxidase complex for 1 h. Finally, the sections were dehydrated by serial rinsing with alcohol, dewaxed in dimethyl and benzene, and cover-slipped. Results were scanned by 3D HISTECH Pannoramic 250, MADE IN Hungary.

### Statistical Analysis

Statistical significance was assessed using one-way ANOVA followed by Fisher's least significant difference (LSD) analysis with *P* < 0.05 as statistically significant. SPSS was used for statistical analysis. Data were expressed as means ± standard error of the mean (SEM).

## Results

### Effects of SNS on Body Weight

Several variations in BW of the male rats were observed from PND 21 to PND 56. The BWs of all male rats were weighed per day from PND 21 after MS. The lower BW was shown in the model group than the control group after MS. The BWs of the MS male rats presented relatively slow weight gain compared with the control group. However, the three groups administered with SNS showed more rapid increase in BW in a dose-dependent manner from PND 42 to PND 56 than model group (Figure 2). MS significantly restrained the increase in weight of these male rats on PND 35. The weight gain of the MS male rats was markedly decreased compared with the control rats from PND 21 to PND 35 (*P* < 0.05). Meanwhile, compared with the model group, the male rats in the three SNS-treated groups gained more weight on PND 35 (*P* < 0.05). In addition, from PND 21 to PND 42, the BWs of the three SNS-treated groups significantly increased compared with the model group (SNS-L and SNS-H, *P* < 0.05; SNS-M, *P* < 0.01). Moreover, no significant differences were observed among the six groups from PND 21 to PND 28, 49, and 56 (Table 1).

### Effects of SNS on Anhedonia

**Figure 3:**
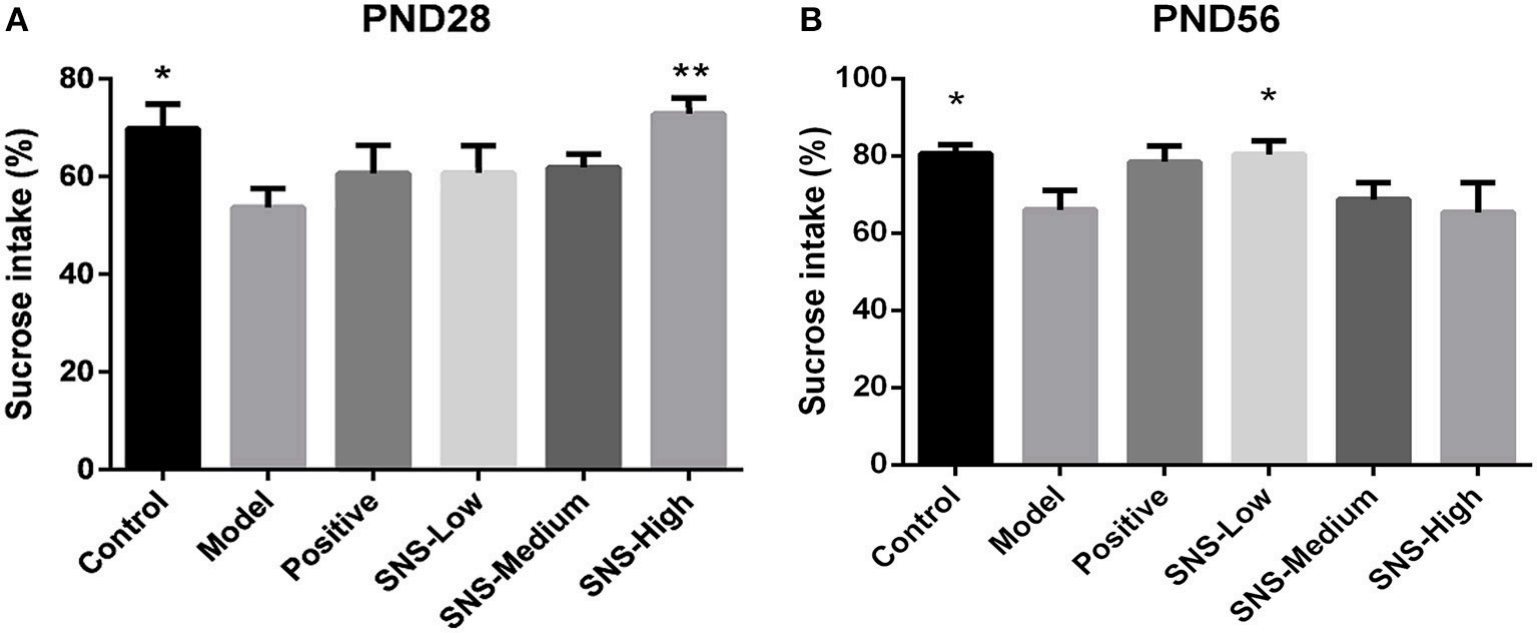
Effects of SNS on the anhedonia of MS male rats during the sucrose preference test (SPT). The animals were examined by the reduction of 1% sucrose consumption on PND 28 **(A)** and PND 56 **(B)**. The values were analyzed by one-way ANOVA with Fisher's least significant difference (LSD) from 6 young rats and 5 adult rats per group. Data are expressed as means ± SEM. *P < 0.05, **P < 0.01 vs. model (statistically significant).

SPT was performed for 2 h on PND 28 and PND 56 for the young and adult stages, respectively, to evaluate depression-like behavior. MS contributed to anhedonia in both stages. SNS-H and SNS-L treatments alleviated the depressive behavior in young and adult MS male rats. Among the young MS male rats, specific consumption of 1% sugar water in the model group was markedly reduced compared with the control group (*P* < 0.05). However, data from the SNS-H group showed significantly reversed results compared with MS rats without treatment (*P* < 0.01) (Figure 3A). Compared with the control group, specific consumption of 1% sugar water of the adult male rats in the model group was significantly decreased (*P* < 0.05). Nevertheless, SNS-L treatment on the MS male rats markedly increased the consumption of 1% sugar water compared with the model group (*P* < 0.05) (Figure 3B).

### Effects of SNS on Behavioral Despair

**Figure 4:**
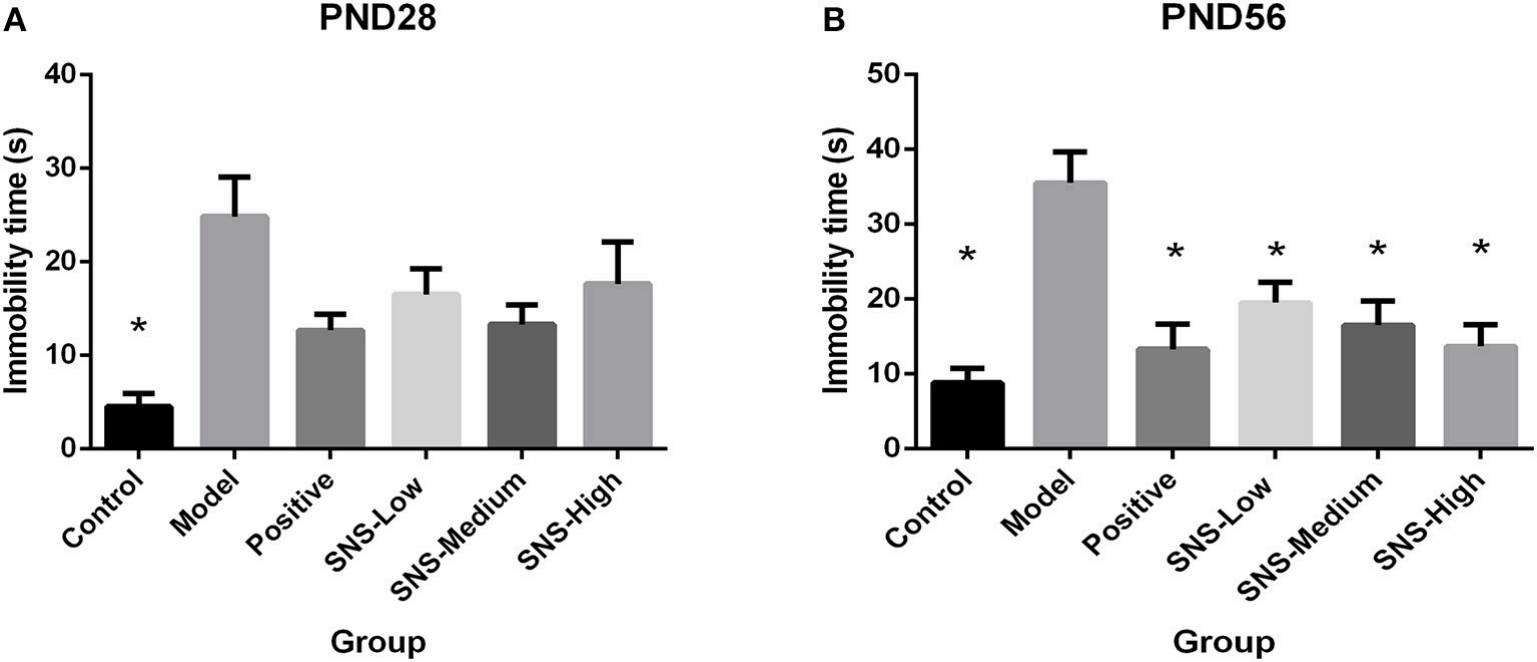
Effects of SNS on the behavioral despair of MS male rats during the forced swimming test (FST). The rats were assessed by immobility time on PND 28 **(A)** and PND 56 **(B)**. Data were analyzed by one-way ANOVA with Fisher's LSD from 6 young rats and 5 adult rats per group and expressed as means ± SEM. *P < 0.05 vs. model (statistically significant).

FST was used to assess the depression-like behaviors on PND 28 and PND 56 for the young and adult stages, respectively. All male rats underwent FST for 5 min after a 15 min pre-test session on the day before. MS induced behavioral despair on both stages. However, medication administration did not affect this depressive behavior during FST for young MS male rats, but relief of behavioral despair was observed for adult MS male rats. On PND 28, the immobility time of the MS male rats in the model group was significantly augmented compared with the control male rats (*P* < 0.05). However, only reduced immobility time was observed in the MS male rats in all drug intervention groups, compared with the MS male rats in the model group, but without statistical significance (Figure 4A). The immobility duration of the adult male rats in the model group was markedly increased compared with that in the control group (*P* < 0.05), but significant decrease in immobility time was observed in the MS male rats in all drug intervention groups (*P* < 0.05) (Figure 4B).

### Effects of SNS on Anxiety-Like Behavior

**Figure 5:**
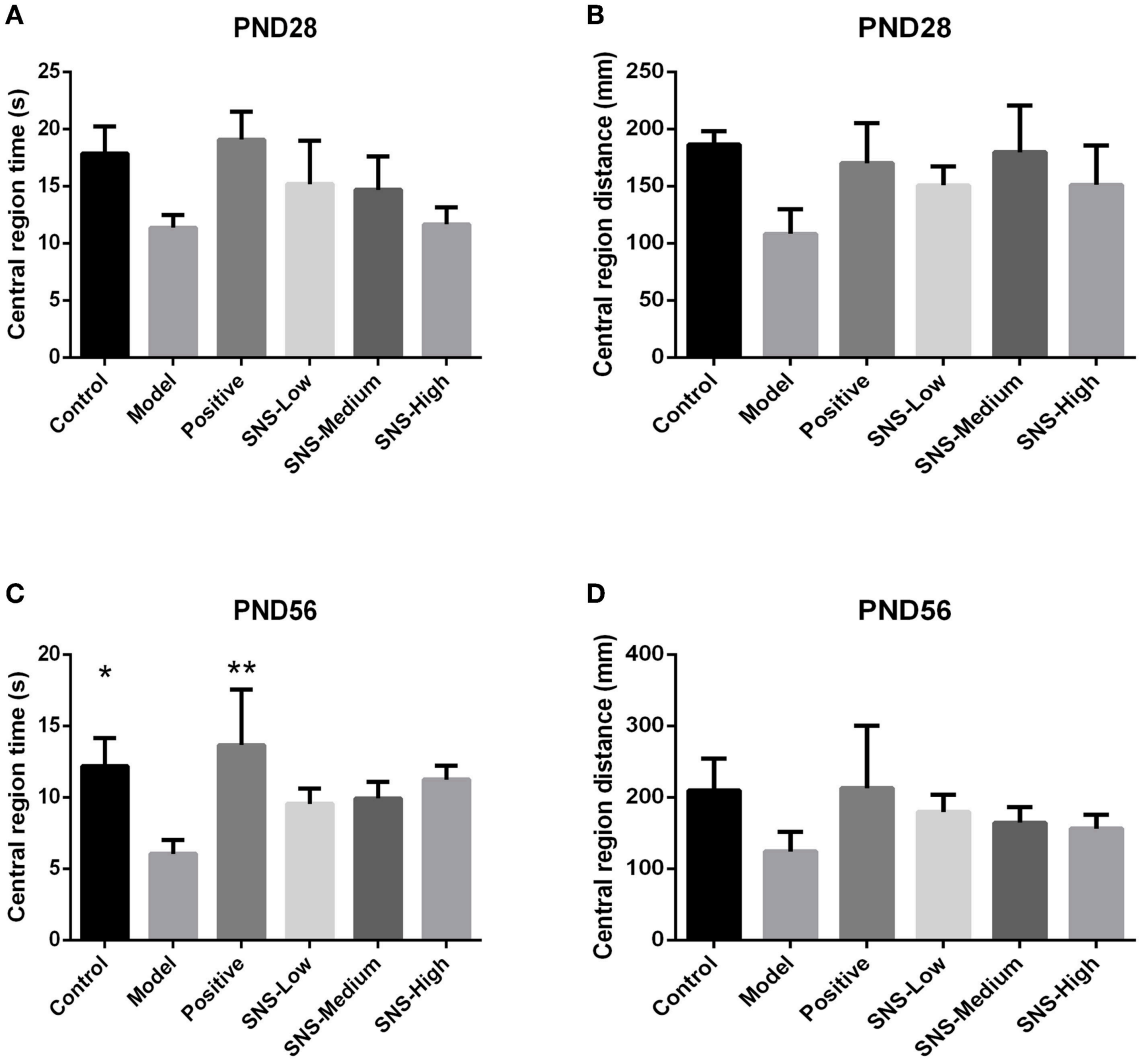
Effects of SNS on the anxiety-like behavior of MS male rats. The animals were evaluated by the time spent at the central region on PND 28 **(A)** and PND 56 **(C)** and the distance within the central region on PND 28 **(B)** and PND 56 **(D)** in the open field test (OFT). Data were analyzed by one-way ANOVA with Fisher's LSD from 5 to 8 rats per group and are shown as means ± SEM. *P < 0.05 and **P < 0.01 vs. model (statistically significant).

OFT was performed on PND 28 and 56 to evaluate the anxiety-like behavior. MS induced anxiety-like behavior in the MS adult male rats during OFT, and fluoxetine mitigated this behavior. The results showed that the central region time and distance of young male rats had no marked differences among the six groups, only a decreasing tendency was observed in the model group vs. the control group and increased in the positive, SNS-L, SNS-M, and SNS-H groups vs. the model group (Figures 5A,B). For the adult male rats during OFT, the MS rats in the model group exhibited significant decline in the central region time compared with the control rats, and comparing with MS rats in model group the data from MS rats treated by fluoxetine were significantly reversed (*P* < 0.05, Figure 5C). However, significant differences were not found in the central region distance in the adult male rats, which only showed a trend similar to that in the central region time (Figure 5D).

### Impact of SNS on 5-HT1A Receptor by Immunohistochemistry

**Figure 6:**
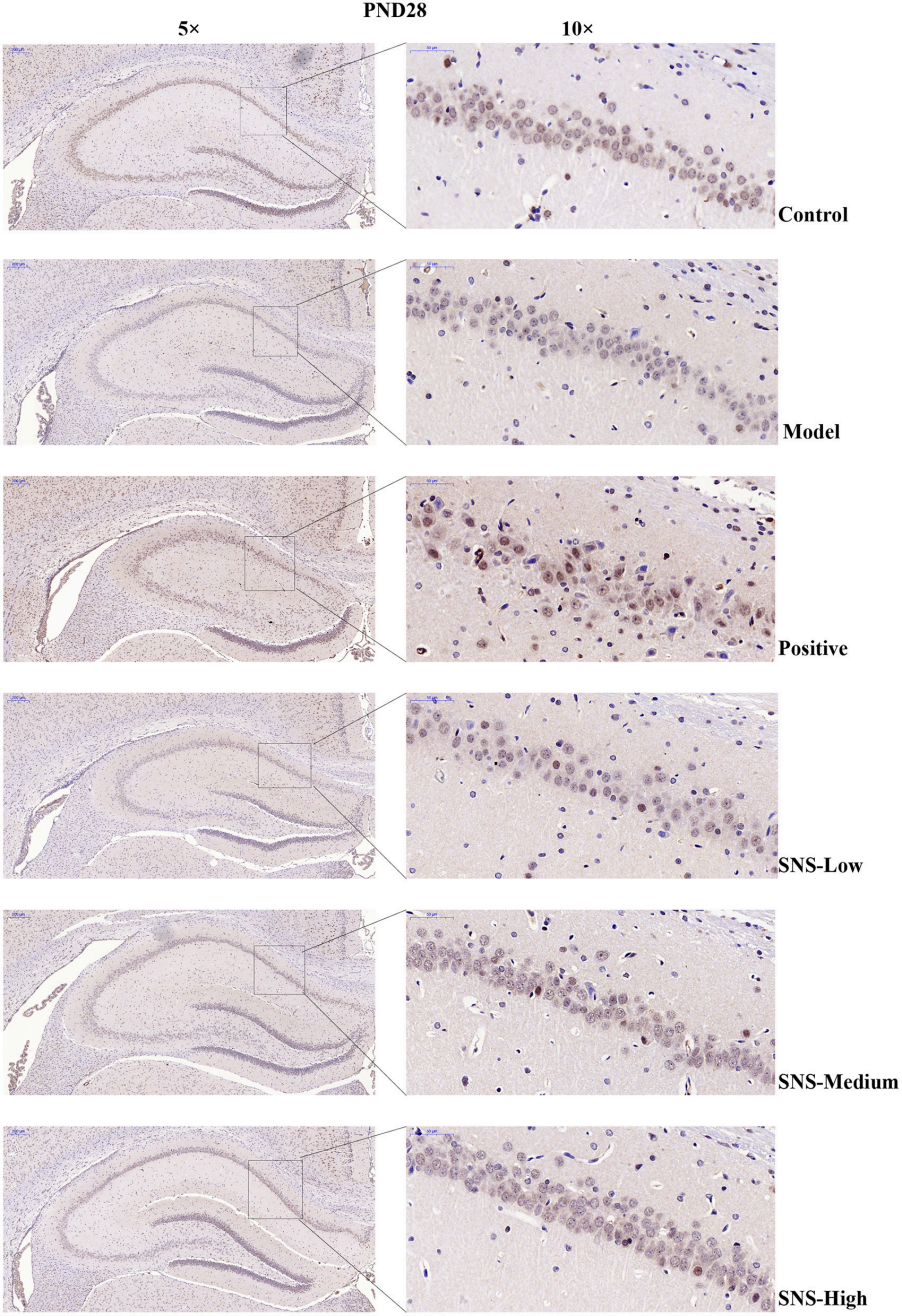
Change in the hippocampal 5-HT1A receptor expression of rats on PND 28 by immunohistochemistry (IHC). The effects of SNS treatment on 5-HT1A receptor expression in the CA1 area of cerebral hippocampus of MS male rats on PND28 as shown by IHC. Scale bar = 50 μm.

**Figure 7:**
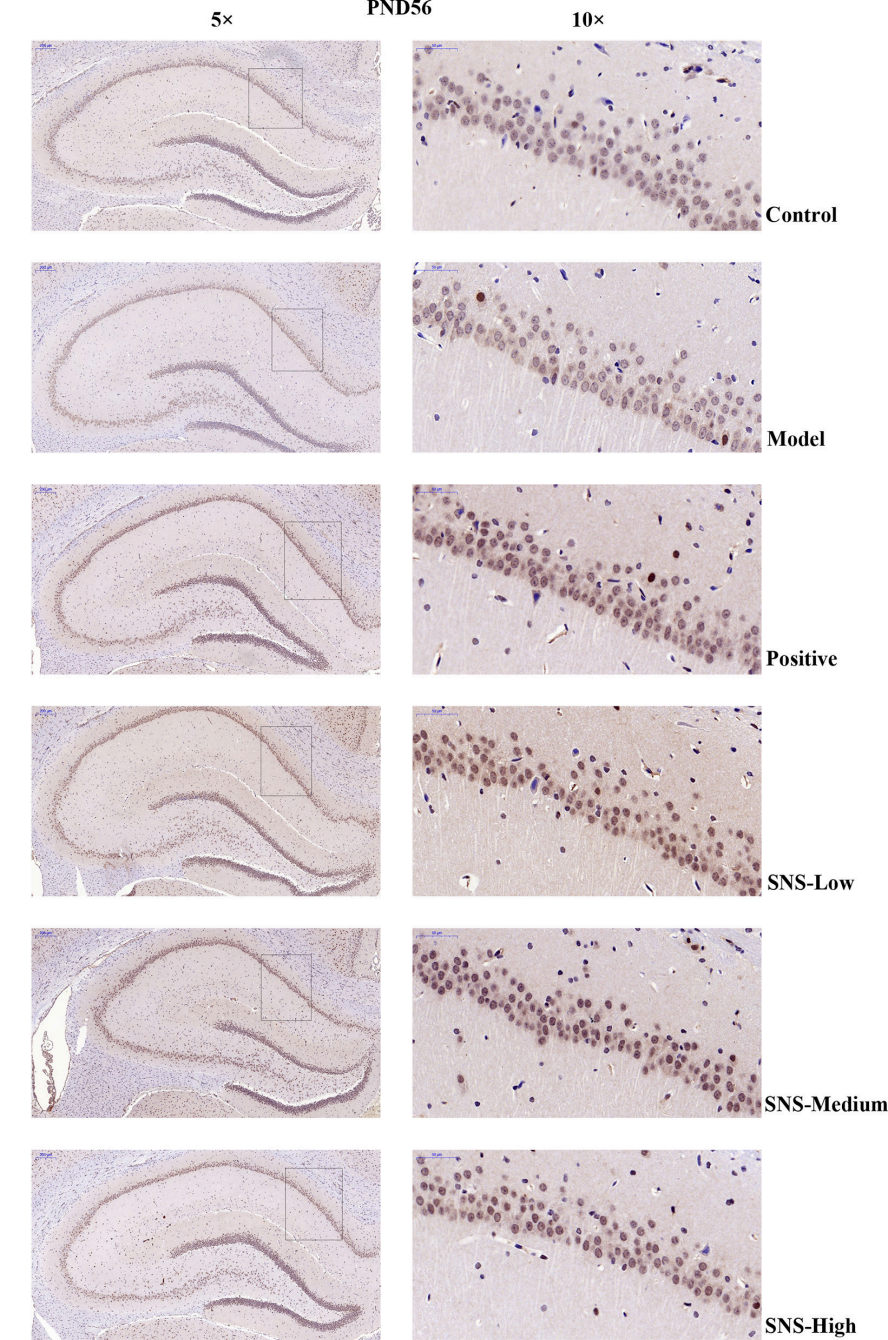
Change in the expression of hippocampal 5-HT1A receptor of rats on PND 56 by IHC. The effects of SNS treatment on 5-HT1A receptor expression in the CA1 area of cerebral hippocampus of MS male rats on PND56 as shown by IHC. Scale bar = 50 μm.

The expression of 5-HT1A receptor in the CA1 region of the hippocampus of the male rats was detected by IHC (Figure 5). MS triggered the decrease in 5-HT1A receptor expression in the CA1 region in the hippocampus of young (PND 28) and adult (PND 56) male rats compared with control rats without MS. However, the expression was upregulated by SNS treatment on MS rats compared with model group. The results show that the expression of 5-HT1A receptor was slightly reduced in the CA1 of hippocampus of the male rats subjected to MS at PND 28 compared with control rats. This phenomenon was reversed by fluoxetine and medium and high SNS doses compared with MS rats but no treatment (Figure 6). On PND 56, the expression of 5-HT1A receptor was obviously downregulated by MS in the CA1 region of hippocampus of adult male rats compared with control group. The downregulation of 5-HT1A receptor expression in the CA1 in the hippocampus of adult male rats subjected to MS was reversed by fluoxetine and SNS treatment compared with model group (Figure 7).

### Impact of SNS on BDNF Signal Pathway at PND28

**Figure 8:**
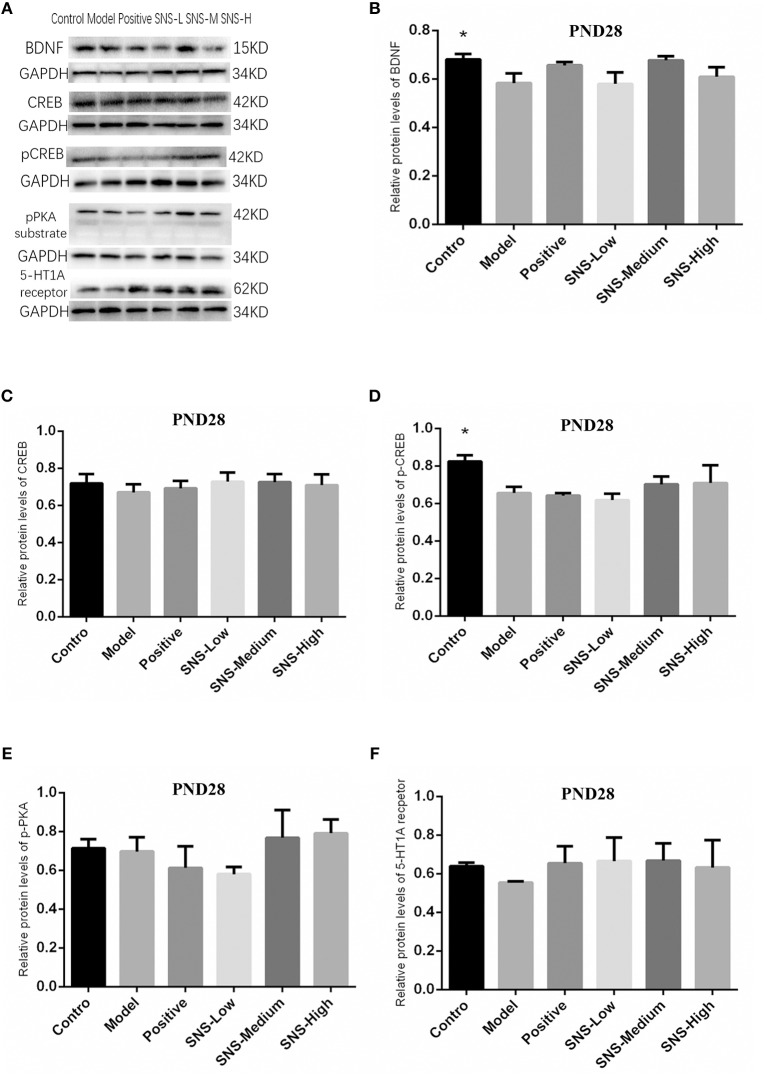
Impacts of SNS on the expression of 5-HT1A receptor/CREB/BDNF in MS rats on PND 28. Impacts of SNS treatments on the 5-HT1A receptor/CREB/BDNF signaling in the cerebral hippocampus of MS male rats on PND 28. The bands of protein in the hippocampus of each group tested by Western blot (WB) are shown in **(A)**. Differences were analyzed by one-way ANOVA with Fisher's LSD. The results are presented as means ± SEM. The relative protein levels of the brain-derived neurotrophic factor (BDNF) **(B)**, cAMP-response element binding protein (CREB) **(C)**, phospho-CREB (pCREB) **(D)**, phospho-protein kinase A (pPKA) substrate **(E)**, and serotonin 1A (5-HT1A) receptor **(F)** in the cerebral hippocampus of young (PND28) male rats of each group (*n* = 4) were detected by WB. *P < 0.05 compared with the model group.

The results indicated that, compared with the young male rats in the control group, the expression of pCREB (Figure 8D) and BDNF (Figure 8B) in the cerebral hippocampus of MS rats was significantly downregulated (*P* < 0.05), but without statistical significance for the medication treatment groups compared with the model group. The expression of 5-HT1A receptor in the cerebral hippocampus of young male rats in the model group was downregulated by MS compared with that in the control group, but fluoxetine and SNS administration reversed this downregulation, compared with the MS rats in the model group. These results are without statistical significance (Figure 8F). Nevertheless, the expression of CREB (Figure 8C) and pPKA substrate in the cerebral hippocampus of adult male rats (Figure 8E) showed no marked differences among the groups. In addition, the bands of these proteins are expressed in Figure 8A.

### Impact of SNS on BDNF Signal Pathway at PND56

**Figure 9:**
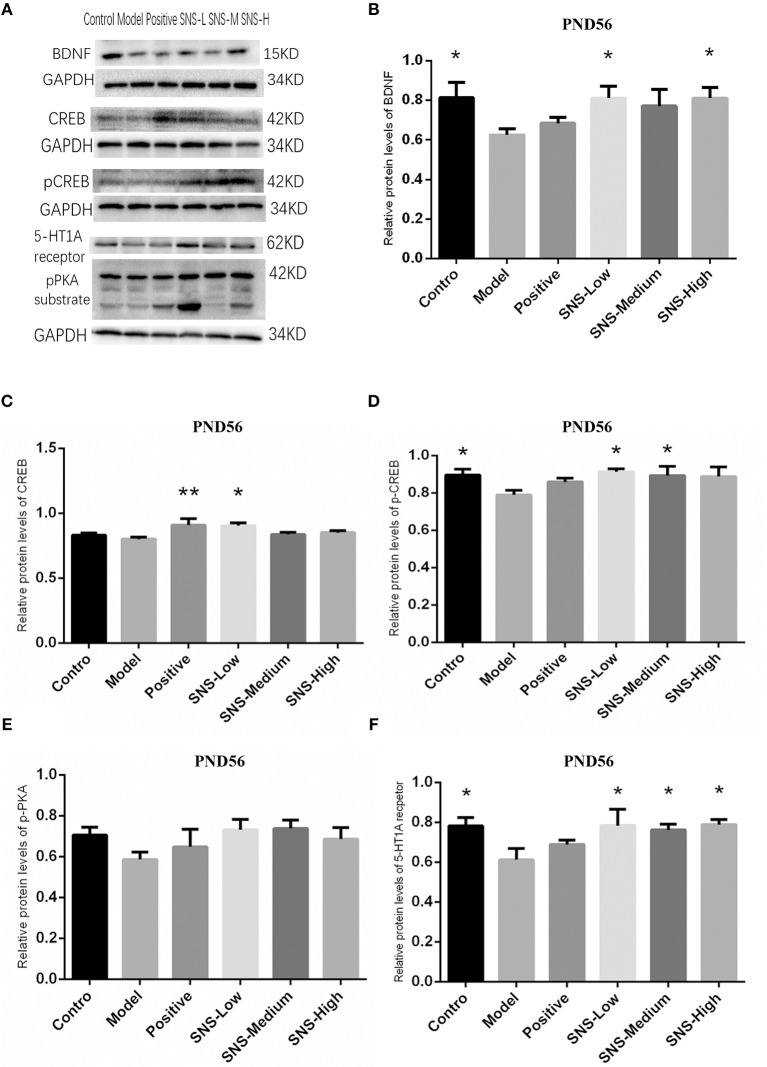
Impacts of SNS on the expression of 5-HT1A receptor/CREB/BDNF in MS rats on PND56. Impacts of SNS treatments on the 5-HT1A receptor/CREB/BDNF signaling in the cerebral hippocampus of MS male rats on PND 56. The bands of protein in the hippocampus of each group tested by WB are shown in **(A)**. Differences were analyzed by one-way ANOVA with Fisher's LSD. The results are shown as means ± SEM. The relative protein levels of BDNF **(B)**, CREB **(C)**, pCREB **(D)**, pPKA substrate **(E)**, and 5-HT1A receptor **(F)** expression in the cerebral hippocampus of adult (PND56) male rats of each group (*n* = 4) were assessed by WB. *P < 0.05, **P < 0.01 compared with the model group.

The results of CREB/BDNF signal pathway of cerebral hippocampus in adult male rats were analyzed (Figure 8). CREB/BDNF signal pathway in the cerebral hippocampus of adult male rats was downregulated by MS compared with control rats, and SNS treatment reversed this downregulation on MS rats compared with the MS rats in model group. The results showed that the expression of 5-HT1A receptor was significantly decreased in the cerebral hippocampus of adult MS male rats in the model group compared with the control group, and SNS treatment markedly reversed the downregulation compared with the MS rats in the model group (Figure 9F). No significant differences were found in the expression of CREB in the cerebral hippocampus of adult male rats between the control and model groups. However, fluoxetine and low SNS dose administration significantly upregulated CREB expression of MS rats compared with MS rats in the model group (*P* < 0.05, *P* < 0.01; Figure 9C). One-way ANOVA demonstrated that the expression of pCREB in the cerebral hippocampus of adult male rats was markedly decreased in the model group compared with control group, which was significantly reversed by low and medium doses SNS compared with the MS rats in model group (*P* < 0.05; Figure 9D). The expression of pPKA substrate in the cerebral hippocampus of adult male rats was downregulated in the MS rats in the model group vs. the control group but upregulated in the MS rats administered with medication vs. the untreated MS rats in model group (Figure 9E). MS markedly reduced the expression of BDNF in the cerebral hippocampus of adult male rats compared with the control rats. Meanwhile, low and medium doses of SNS significantly upregulated the BDNF expression compared with the MS rats in the model group (*P* < 0.05; Figure 9B). All protein bands are shown in Figure 9A.

## Discussion

In present study, we manifested several attractive findings on the antidepressant effect of SNS treatment from the results of behavior and molecular level in depressive rats. The MS paradigm used for the animal model of depression in this study has been considered as a classic depressive model for early life stress on rats (22). Our study was aimed to illustrate the effects of SNS on depression-like behavior of both young and adult rats that experienced MS during the postnatal period. Moreover, we further detected the relative proteins of potential signaling pathway involved in the mechanism of depressive symptoms and the antidepressant effect of SNS. Thus, the results might present a new discovery that may guide the clinical medication for new therapy for depression caused by trauma during childhood.

### Antidepressant Effect of SNS

In our study, the BW was measured from PND 21 pre-treatment and throughout the whole experiment. The weights of the MS rats presented a lower trend than normal rats and MS rats administered with SNS from PND 21 to PND 56. However, fluoxetine exerted no effects on weight in MS rats (Figure 2). From the BW gain of rats with the baseline at PND 21, MS reduced the BW gain at PNDs 28, 35, 42, 49, and 56, but SNS treatment reversed this process. However, compared with the MS group, the fluoxetine group exhibited no increasing trend in the BW gain, although significant statistical difference was only observed on PND 35 (Table 1). The results showed that SNS could improve the loss of weight in MS rats during the growth stage until adulthood, which showed that the MS rats could develop some symptoms, such as eating less and losing weight, whereas SNS might ameliorate this appetite and nutrition condition in MS rats. The result on weight was consistent with several studies which have indicated that the postnatal MS could reduce the weight gain compared with normal breast-feeding in rats and caused under nutrition accompanied with the damage to insulin-like growth factor-1 (IGF-1) levels and growth and alteration of the hippocampus (23, 24). Meanwhile, a recent study manifested the effect of SNS on the psychological stress and stress-related nonalcoholic fatty liver disease. The results indicated that the SNS group administered with long-term chronic restraint stress showed a significant increase in BW gain compared with the stress group (25). However, the absence of effect of fluoxetine on the loss of weight in MS rats may be attributed to the enhanced satiety for food and restrain hunger-related pathways, as previously reported (26).

SPT and FST are usually applied to rats to evaluate depression-like behavior and antidepressant effect. The effect of SNS on mental disorders and chronic stress-related diseases, such as depression, has been already demonstrated in some studies, but age specificity was not mentioned (27, 28). Our results indicated that young and adult MS rats exhibited anhedonia during SPT as shown by the reduced sucrose consumption and behavioral despair during FST as shown by augmented immobility time. The effect of fluoxetine and SNS on depression-like behavior could be observed in young (PND 28) and adult (PND 56) MS rats. Fluoxetine and SNS treatment resulted in increased sucrose consumption during SPT and reduced immobility time during FST, when compared with the untreated MS rats. For the young rats, only high SNS dose significantly improved anhedonia during SPT, which was observed in the low dose group for adult rats (Figure 3). During FST, although all SNS and fluoxetine groups showed significantly higher immobility time compared with the MS group in adult rats, SNS and fluoxetine increased the immobility time of young MS rats but without statistical significant difference (Figure 4). However, no significant difference was found between the SNS and fluoxetine groups in all rats. These findings illustrated that SNS could take effect on depression-like behaviors depending on the doses and duration of treatment for rats that experienced early life stress, such as MS. In addition, the reason why the effect of fluoxetine on depression-like behaviors only emerged in adult rats presented by FST might due to its side-effect, such as delayed onset time (29). Nevertheless, only a few studies have focused on the onset time of SNS treatment on mental disorders, especially for emotional diseases induced by early life stress. This investigation is needed to be the next step to provide evidence for clinical medication. In addition, no statistical differences were found on the time and distance spent during OFT between MS and normal rats for young rats but with decreasing trend. However, for adult rats, the time, rather than distance spent, at the central area were significantly decreased by MS. Meanwhile, although SNS treatment increased the time and distance spent at the central area in the young and adult rats, no statistical difference was found in the improvement of anxious symptom for the fluoxetine group, which showed marked increase in these two indicators (Figure 5) During OFT, no statistical differences were found probably because of the insufficient sample size. Therefore, our results during OFT could indicate that the impact of MS on anxiety-like behavior might emerge from the young stage to the adult stage, and SNS could alleviate this symptom to some extent. Only a few studies have focused on the effect of SNS to mitigate the depression-like behavior of young rats. Nevertheless, a previous study reported that modified SNS treatment (from PND 24 to PND 51) relatively improved the depression-like behavior of adolescent rats maternally deprived from PND 1 to PND21, followed by chronic unpredictable stress from PND 24 to PND 51. The improvement was observed in weight assessment, SPT, OFT, and the level of hypothalamic pituitary adrenal (HPA) axis (30). Similar to the present study, several previous studies illustrated the antidepressant effect of SNS and its active ingredients on chronic unpredictable stress rats expressed by behavioral tests, such as SPT, FST, OFT, or tail suspension test, during adulthood. SNS could improve anhedonia, behavioral despair, and anxiety-like behavior of adult rats (31–33). Therefore, SNS may be used to treat emotional diseases, especially the depression caused by early life adversity.

### Role of PKA/CREB/BDNF Pathway on Antidepressant of SNS

The BDNF plays an important role on neurogenesis, neuroplasticity, and nerve cell survival in the hippocampus, which is closely associated with the pathogenesis of depression and involved in the mechanism of antidepressant (34–36). Previous studies showed that the PKA-CREB-BDNF pathway and the phosphorylation of PKA and CREB proteins in the hippocampus could be downregulated by CUMS, whereas the expression of these proteins was normalized by traditional Chinese prescription or its active ingredient (37). Moreover, the role of 5-HT1A receptor, as a crucial receptor of serotonin system regarded as the major mechanism of mood modification under stress, has been previously reported. The 5HT1A receptor played an important role in the neurogenesis and synapse formation probably by activating certain signaling pathways and may be linked to a certain deficit in behavior and cognition (38). Thus, we paid attention to the alterations in 5-HT1AR and BDNF, as well as the relative protein on the relative pathway (including the pPKA substrate, CREB, and pCREB), influenced by SNS treatment on MS-induced depressive rats. In the present study, SNS administration influenced the 5-HT1A receptor/pPKA substrate/CREB/BDNF signaling pathway in the cerebral hippocampus of MS rats on PND 28 (young) and PND 56 (adult) as shown by the measured expression of the 5HT1A receptor, pPKA substrate, CREB, pCREB, and BDNF protein.

First, at PND 28, the IHC results showed that the expression of 5-HT1A receptor in the hippocampal CA1 area was reduced by postnatal MS but reversed by fluoxetine and high dose SNS (Figure 6). Moreover, MS also downregulated the hippocampal expression of the 5-HT1A receptor, CREB, pCREB, and BDNF protein. However, statistical significant difference was found only on pCREB and BDNF, compared with the control rats, as shown by the WB test. Although SNS upregulated the hippocampal expression of 5-HT1A receptor, CREB, pCREB and BDNF protein, compared with untreated MS rats, no significant differences were observed among these proteins (Figure 8). One previous study suggested that MS could impair the synaptogenesis with decreased spine formation and maturation during the early development of brain in postnatal period. This phenomenon may be mediated by downregulating the BDNF/ERK signaling pathway and may produce certain impairment of hippocampal-dependent learning ability (39). The impairment of MS on the function of cerebral hippocampus, which is as a crucial region involving the mechanism of depression, may emerge during the early development of rats. Thus, the evidence provided in (39) is consistent with our findings, that is, postnatal MS could impair the pCREB/BDNF expression in the hippocampus of MS-induced depressive rats emerging in early development. Thus, during the young stage, MS reduced the hippocampal phosphorylation of CREB such that enough transcription of BDNF gene could not be activated. Thus, the depression-like behavior caused by postnatal MS accompanied alterations in the BDNF upstream signaling pathway in young rats. However, although SNS or fluoxetine treatment did not significantly mitigate the 5HT1A receptor-pPKA substrate-CREB-pCREB-BDNF pathway in the hippocampus of MS rats, compared with the model group, medium and high SNS doses partly restored the expression of 5-HT1A receptor, CREB, pCREB, and BDNF (Figure 8). Only a few studies have investigated the antidepressant effect of SNS on the BDNF relative signaling pathway for MS-induced depressive rats during young stage at PND 28. The present results on young rats may indicate that the impairment of 5HT1A receptor-pPKA substrate-CREB-pCREB-BDNF pathway could not be completely recovered by SNS and fluoxetine for only 1 week treatment, although the depressive symptoms were alleviated by high SNS dose for a week. This result could account for the delayed onset of the antidepressant drug for at least 2 weeks of drug administration. This phenomenon was also reported by a previous study on the effect of active components in SNS for chronic stress induced-depression (40).

**Figure 10:**
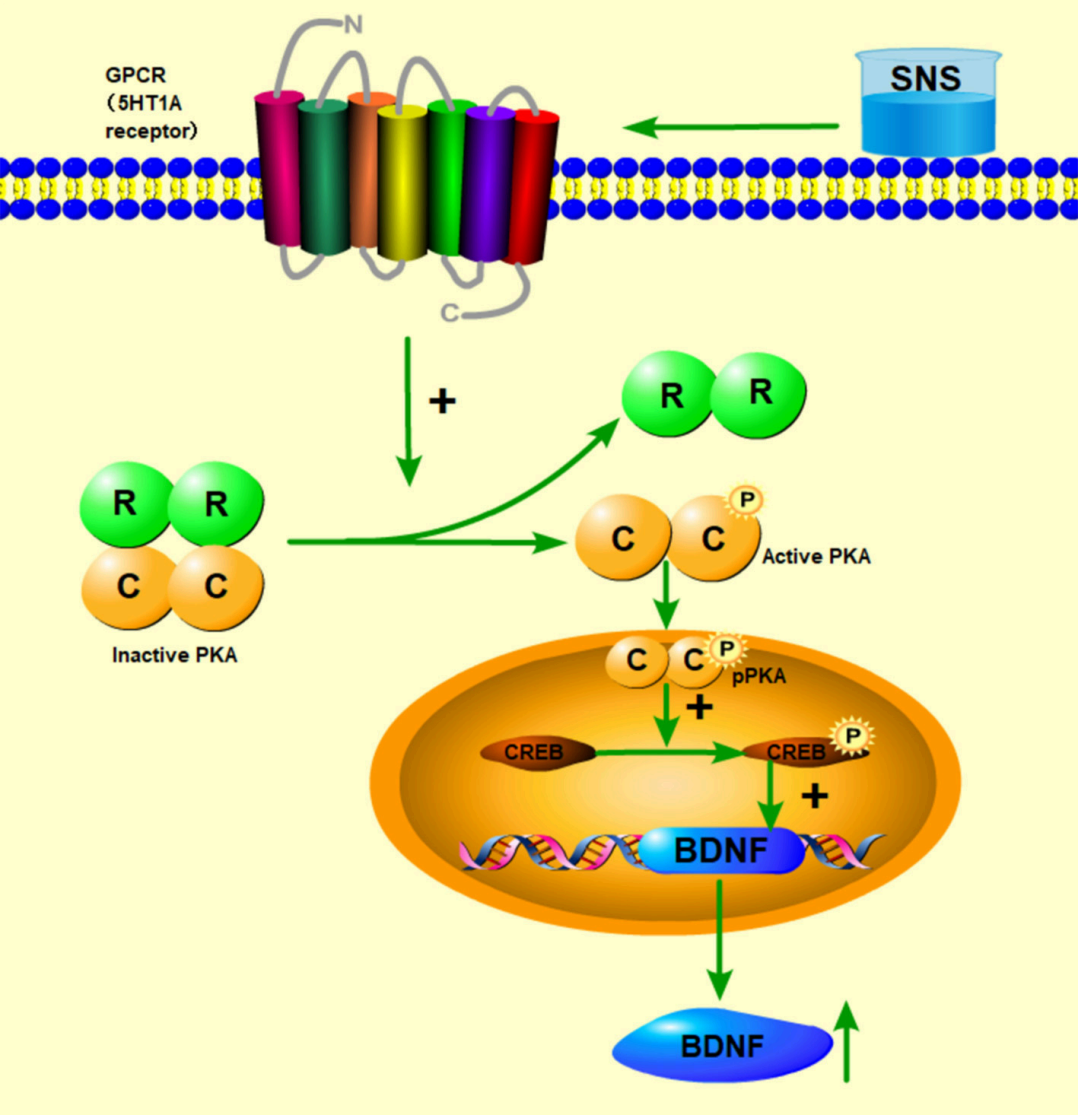
Abridged general view of the potential pathway for the effect of SNS. The mechanism of antidepressant effect for SNS is illustrated. SNS upregulated the 5-HT1A receptor expression, which in turn activated the phosphorylation of PKA. The activation of pPKA mediated the phosphorylation of CREB. Then, pCREB transcribed the expression of the BDNF gene, upregulated the production of BDNF to benefit the hippocampal neurogenesis, and alleviated the mental disorders closely related to the hippocampus.

Then, up to the adulthood of rats at PND 56, the expression of 5-HT1A receptor in the hippocampal CA1 area was downregulated by MS during early life but reversed by fluoxetine and SNS treatment in IFC (Figure 7). Furthermore, we found that 5-HT1A receptor, phosphor-CREB, and BDNF expression were significantly downregulated by postnatal MS in adult rats, and the expression of CREB and pPKA substrate was reduced only with downtrend compared with the non-MS group, as shown by WB analysis. After drug treatment for 5 weeks, SNS significantly restored the expression of 5-HT1A receptor, CREB, pCREB, and BDNF expression and slightly upregulated the pPKA substrate expression in the MS adult rats but without dose specificity. In addition, fluoxetine slightly upregulated these proteins, except for CREB with significant difference compared with the model rats (Figure 9). Thus, MS during early life could generate long-term impacts on the expression of upstream relative protein of BDNF in the hippocampus of rats during adulthood, which coincided with their depression-like behavior. SNS treatment on the MS rats may improve the pCREB/BDNF pathway, which is activated by increased 5-HT1A receptor for adult rats that experienced early life stress. A recent study has illustrated that CREB/BDNF signaling pathway was involved in the mechanism of antidepressant effect. The increased ratio of p-CREB/CREB and mBDNF/proBDNF expression in the hippocampus might contribute to the therapeutic effects on the lipopolysaccharide-intervention rats (41). In addition, similar to the present results, the results from an assay showed that MS could trigger the depression-like behavior accompanied with downregulated CREB/BDNF expression and mRNA level of BDNF (42). Moreover, several studies have stated that antidepressant administration, including physical and Chinese herbs, mitigated depressive behavior by increasing 5HT1A receptor-mediated cAMP/PKA/CREB pathway in the hippocampus of rats that underwent chronic stress for adults, in accordance with our results on MS adult rats (43, 44). A previous research on the effect of SNS treatment on the depression caused by chronic stress also showed that the SNS administration group had significantly higher expression of 5-HT1A receptor than the model group (45). *In vitro* study on the antidepressant mechanism demonstrated that Jiawei SNS exhibited nerve protection and antidepressant effect by upregulating the expression of CREB and pCREB on PC12 cell stressed by corticosterone and glutamate (46). Therefore, our results indicated that the depression-like behaviors in the young and adult rats which experienced early life stress could be alleviated different doses of SNS by regulating the 5-HT1A receptor/CREB/BDNF signaling pathway. However, whether pPKA substrate could be significantly altered under MS and SNS treatment could not be observed just with the trend on PND 56, probably because the antibody we applied was a substrate protein instead of the original pPKA. The substrate protein of pPKA contained the pCREB that was activated by pPKA. Therefore, to some degree, the significant change on pCREB may reflect the effect of MS/SNS on the pPKA substrate. This phenomenon needs further verification by co-immunoprecipitation in our next study. The above results show that the antidepressant effect of SNS on maternal separation rats may work through upregulating the 5-HT1A receptor expression which in turn activated the phosphorylation of PKA. The activation of pPKA mediated the phosphorylation of CREB. Then, pCREB transcribed the expression of the BDNF gene, upregulated the production of BDNF to benefit the hippocampal neurogenesis, and alleviated the mental disorders closely related to the hippocampus (Figure 10).

## Conclusion

In summary, our study still needs to further expound the mechanism of onset time and concentration of SNS on MS models, especially on the pPKA/CREB/BDNF pathway mediated by 5-HT1A receptor and the impacts on the hippocampal neurogenesis. The results showed the positive effects of SNS on the MS-induced depression-like behavior. The present study elucidated the antidepressant effect of traditional Chinese medicine SNS for depression, which may be used for young and adult individuals who have experienced adversity in early life. Importantly, our data first illustrated that the regulation of the hippocampal 5-HT1A receptor/CREB/BDNF pathway may contribute to the antidepressant effect of SNS. Therefore, our findings may provide evidence and clinical guidance to formulate an effective therapy for children and adults undergoing depression caused by stress during early life adversity.

## Data Availability

The datasets are available on request. The raw data supporting the conclusions of this manuscript will be made available by the authors, without undue reservation, to any qualified researcher.

## Ethics Statement

All experimental procedures were followed the guidelines of the International Association for the Use of Animals in Research and approved by the Committee of Animal experiment ethics review in Guangzhou University of Chinese Medicine.

## Author Contributions

YS: designed the study; KC, CS, SB, LY, YY, and LG: performed the experiments; KC and RZ: analyzed the data; KC: wrote the manuscript.

### Conflict of Interest Statement

The authors declare that the research was conducted in the absence of any commercial or financial relationships that could be construed as a potential conflict of interest.

## References

[1] Brodsky BS Oquendo M Ellis SP Haas GL Malone KM Mann JJ. The relationship of childhood abuse to impulsivity and suicidal behavior in adults with major depression. Am J Psychiatry. (2001) 158:1871–7. 10.1176/appi.ajp.158.11.1871 11691694

[2] Teicher MH. Childhood trauma and the enduring consequences of forcibly separating children from parents at the United States border. BMC Med. (2018) 10.1186/s12916-018-1147-y 30131056 PMC6103973

[3] Hall FS. Social deprivation of neonatal, adolescent and adult rats has distinct neurochemical and behavioral consequences. Crit Rev Neurobiol. (1998) 12:129–62. 10.1615/CritRevNeurobiol.v12.i1-2.50 9444483

[4] Amini-Khoei H Mohammadi-Asl A Amiri S Hosseini MJ Momeny M Hassanipour M. Oxytocin mitigated the depressive-like behaviors of maternal separation stress through modulating mitochondrial function and neuroinflammation. Prog Neuropsychopharmacol Biol Psychiatry. (2017) 10.1016/j.pnpbp.2017.02.022 28259722

[5] Zhang Y Zhu X Bai M Zhang L Xue L Yi J. Maternal deprivation enhances behavioral vulnerability to stress associated with miR-504 expression in nucleus accumbens of rats. PLoS ONE. (2013) 8:e69934. 10.1371/journal.pone.0069934 23922862 PMC3724734

[6] Bondar NP Lepeshko AA Reshetnikov VV. Effects of early-life stress on social and anxiety-like behaviors in adult mice: sex-specific effects. Behav Neurol. (2018) 2018:1538931. 10.1155/2018/1538931 29619126 PMC5818933

[7] Oreland S Nylander I Pickering C. Prolonged maternal separation decreases granule cell number in the dentate gyrus of 3-week-old male rats. Int J Dev Neurosci. (2010) 28:139–44. 10.1016/j.ijdevneu.2009.12.005 20079421

[8] Baumgarten HG Grozdanovic ZŽ Anatomy of central serotoninergic projection systems. In: Van de Kar LDŽ editor. Serotoninergic Neurons and 5-HT Receptors in the CNS. New York, NY: Springer (1997) pp. 41–89.

[9] Lambás-Señas L Mnie-Filali O Certin V Faure C Lemoine L Zimmer L Functional correlates for 5-HT1A receptors in maternally deprived rats displaying anxiety and depression-like behaviors. Prog Neuro-Psychopharmacol Biol Psychiatry. (2008) 33:262–8. 10.1016/j.pnpbp.2008.11.017 19111592

[10] Wang Q Shao F Wang W. Maternal separation produces alterations of forebrain brain-derived neurotrophic factor expression in differently aged rats. Front Mol Neurosci. (2015). 8:49. 10.3389/fnmol.2015.00049 26388728 PMC4555027

[11] Murínová J Hlaváčová N Chmelová M Riečanský I. The evidence for altered BDNF expression in the brain of rats reared or housed in social isolation: a systematic review. Front Behav Neurosci. (2017) 11:101. 10.3389/fnbeh.2017.00101 28620285 PMC5449742

[12] Mattson MP Maudsley S Martin B. BDNF and 5-HT: a dynamic duo in age-related neuronal plasticity and neurodegenerative disorders. Trends Neurosci. (2004) 27:589–94. 10.1016/j.tins.2004.08.001 15374669

[13] Wang H Zhao Y Wang YJ Song L Wang JL Huang C. Antidepressant- like effects of tetrahydroxystilbene glucoside in mice: involvement of BDNF signaling cascade in the hippocampus. CNS Neurosci Ther. (2017) 23:627–36. 10.1111/cns.12708 28547794 PMC6492667

[14] Liao L Zhang XD Li J Zhang ZW Yang CC Rao CL. Pioglitazone attenuates lipopolysaccharide-induced depression-like behaviors, modulates NF-κB/IL-6/STAT3, CREB/BDNF pathways and central serotonergic neurotransmission in mice. Int Immunopharmacol. (2017) 49:178–86. 10.1016/j.intimp.2017.05.036 28595081

[15] Pliakas AM Carlson RR Neve RL Konradi C Nestler EJ Carlezon WA. Altered responsiveness to cocaine and increased immobility in the forced swim test associated with elevated cAMP response element-binding protein expression in nucleus accumbens. J Neurosci. (2001) 21:7397–403. 10.1523/JNEUROSCI.21-18-07397.2001 11549750 PMC4205577

[16] Wang C Guo J Guo R. Effect of XingPiJieYu decoction on spatial learning and memory and cAMP-PKA-CREB-BDNF pathway in rat model of depression through chronic unpredictable stress. BMC Complement Altern Med. (2017) 17:73. 10.1186/s12906-016-1543-9 28118829 PMC5260079

[17] Uher R Mors O Rietschel M Rajewska-Rager A Petrovic A Zobel A. Early and delayed onset of response to antidepressants in individual trajectories of change during treatment of major depression: a secondary analysis of data from the Genome-Based Therapeutic Drugs for Depression (GENDEP) study. J Clin Psychiatry. (2011) 72:1478. 10.4088/JCP.10m06419 22127194

[18] Trivedi MH Rush AJ Wisniewski SR Nierenberg AA Warden D Ritz L. Evaluation of outcomes with citalopram for depression using measurement based care in STAR^*^D: implications for clinical practice. Am J Psychiatry. (2006)163:28–40. 10.1176/appi.ajp.163.1.28 16390886

[19] Junling L Kai X Tian W Xiaoxing X Jinglu Y Huihui Z Current progress of Sini powder in depression. Glob Tradit Chin Med. (2017) 1:1 10.3969/J.issn.1674-1749.2017.01.038

[20] Willner P Towell A Sampson D Sophokleous S Muscat R. Reduction of sucrose preference by chronic unpredictable mild stress, and its restoration by a tricyclic antidepressant. Psychopharmacology. (1987) 93:358–64. 10.1007/BF00187257 3124165

[21] Detke MJ Lucki I. Detection of serotonergic and noradrenergic antidepressants in the rat forced swimming test: the effects of water depth. Behav Brain Res. (1996) 73:43–6. 10.1016/0166-4328(96)00067-8 8788475

[22] Tractenberg SG Levandowski ML de Azeredo LA Orso R Roithmann LG Hoffmann ES (2016) An overview of maternal separation effects on behavioral outcomes in mice: evidence from a four-stage methodological systematic review. Neurosci Biobehav Rev. 68:489–503. 10.1016/j.neubiorev.2016.06.021 27328784

[23] de Almeida Magalhães T Correia D de Carvalho LM Damasceno S Brunialti Godard AL. Maternal separation affects expression of stress response genes and increases vulnerability to ethanol consumption. Brain Behav. (2018) 8:e00841. 10.1002/brb3.841 29568676 PMC5853632

[24] Figueiredo ÍL Frota PB da Cunha DG da Silva Raposo R Canuto KM de Andrade GM. Prolonged maternal separation induces undernutrition and systemic inflammation with disrupted hippocampal development in mice. Nutrition. (2016) 32:1019–27. 10.1016/j.nut.2016.02.016 27157468 PMC4967409

[25] Cheng F Ma C Wang X Zhai C Wang G Xu X. Effect of traditional Chinese medicine formula SiNiSan on chronic restraint stress-induced nonalcoholic fatty liver disease: a rat study. BMC Complement Altern Med. (2017) 17:203. 10.1186/s12906-017-1707-2 28388904 PMC5383977

[26] da Silva AI Braz GRF Silva SCA Pedroza AADS de Lima-Júnior NC Silva TLA. Body composition, biochemical, behavioral and molecular alterations in overfed rats after chronic exposure to SSRI. Behav Brain Res. (2018) 356:62–70. 10.1016/j.bbr.2018.08.007 30099029

[27] Shen X Zhao Z Luo X Wang H Hu B Guo Z. Systems pharmacology based study of the molecular mechanism of SiNiSan formula for application in nervous and mental diseases. Evid Based Complement Alternat Med. (2016) 2016:9146378. 10.1155/2016/9146378 28058059 PMC5183803

[28] Wang YT Tan QR Sun LL Cao J Dou KF Xia B. Possible therapeutic effect of a Traditional Chinese medicine, SiNiSan, on chronic restraint stress related disorders. Neurosci Lett. (2009) 449:215–9. 10.1016/j.neulet.2008.10.100 19007859

[29] Iñiguez SD Alcantara LF Warren BL Riggs LM Parise EM Vialou V. Fluoxetine exposure during adolescence alters responses to aversive stimuli in adulthood. J Neurosci. (2014) 34:1007–21. 10.1523/JNEUROSCI.5725-12.2014 24431458 PMC3891944

[30] Guo LL Shi YF Sang F Yuan YM Wu HM Effect of maternal deprivation following with chronic unpredictable stress on behavior and HPA axis in adolescent rats and intervention of modified Sini San. Trad Chinese Drug Res Clin Pharmacol. (2016) 27:4 10.1937/jissn.1003-9783.2016.04.004

[31] Chang HS Liang JC Sun JN Wang QG Li J Effect of effective fraction of Sini powder on mouse model of behavioral despair and medical depression. J Beijing Univ Traditional Chinese Med. (2006) 29:7.

[32] Chang HS Wang QG Shi RB Jin Yan Effects of the active components of Sini powder on the behavior and cerebral monoamine neurotransmitters in the rat model of depressive chronic stress. J Beijing Univ TCM. (2003) 26:5.

[33] Li M Xu XD Effect of Sini Powder on behavior and neurotransmitter of rat with chronic stress-induced depression. J Changchun Univ Tradit Chin Med. (2014) 30:8 10.13463/j.cnki.cczyy.2014.04.006

[34] Djalali S Höltje M Grosse G Rothe T Stroh T Grosse J. Effects of brain-derived neurotrophic factor (BDNF) on glial cells and serotonergic neurones during development. J Neurochem. (2005) 92:616–27. 10.1111/j.1471-4159.2004.02911.x 15659231

[35] Phillips C. Brain-derived neurotrophic factor, depression, and physical activity: making the neuroplastic connection. Neural Plast. (2017) 2017:7260130. 10.1155/2017/7260130 28928987 PMC5591905

[36] Björkholm C Monteggia LM. BDNF - a key transducer of antidepressant effects. Neuropharmacology. (2016) 102:72–9. 10.1016/j.neuropharm.2015.10.034 26519901 PMC4763983

[37] Liu Z Qi Y Cheng Z Zhu X Fan C Yu SY. The effects of ginsenoside Rg1 on chronic stress induced depression-like behaviors, BDNF expression and the phosphorylation of PKA and CREB in rats. Neuroscience. (2016) 322:358–69. 10.1016/j.neuroscience.2016.02.050 26926964

[38] Rojas PS What do we really know about 5-HTReceptor signaling in neuronal cells? Front Cell Neurosci. (2016) 10:272 10.3389/fncel.2016.00272 27932955 PMC5121227

[39] Ohta KI Suzuki S Warita K Kaji T Kusaka T Miki T. Prolonged maternal separation attenuates BDNF-ERK signaling correlated with spine formation in the hippocampus during early brain development. J Neurochem. (2017) 141:179–94. 10.1111/jnc.13977 28178750

[40] Jin Y Wang QG Shi RB Influence of active components of Sini powder on the expression of 5-HT1A receptor mRNA in the cortex and hippocampus of the rat model of depression induced by chronic stressors. J Beijing Univ TCM. (2004) 27:4.

[41] Li DD Xie H Du YF Long Y Reed MN Hu M. Antidepressant-like effect of zileuton is accompanied by hippocampal neuroinflammation reduction and CREB/BDNF upregulation in lipopolysaccharide-challenged mice. J Affect Disord. (2018) 227:672–80. 10.1016/j.jad.2017.11.047 29174741

[42] Bian Y Yang L Wang Z Wang Q Zeng L Xu G. Repeated three-hour maternal separation induces depression-like behavior and affects the expression of hippocampal plasticity-related proteins in C57BL/6N mice. Neural Plast. (2015) 2015:627837. 10.1155/2015/627837 26798520 PMC4700195

[43] Kim MH Leem YH. Chronic exercise improves repeated restraint stress-induced anxiety and depression through 5HT1A receptor and cAMP signaling in hippocampus. J Exerc Nutrition Biochem. (2014) 18:97–104. 10.5717/jenb.2014.18.1.97 25566444 PMC4241932

[44] Wang XL Gao J Wang XY Mu XF Wei S Xue L. Treatment with Shuyu capsule increases 5-HT1AR level and activation of cAMP-PKA-CREB pathway in hippocampal neurons treated with serum from a rat model of depression. Mol Med Rep. (2018) 17:3575–82. 10.3892/mmr.2017.8339 29286104 PMC5802157

[45] Yu Q Jin GL Impacts of three Chinese herbal compound on the 5-HT1A receptor gene expression of liver qi stagnation model rat induced by chronic stress. Beijing J Tradit Chin Medi. (2008). 27:1 10.16025/J.1674-1307.2008.01.030

[46] Yan C Wu LL Pan Y Song Q Ran CL Liu SK The effect of Jiaweisinisan on cAMP response element binding protein and phosphorylation in PC12 cells injured by Corticosterone and Glutamate. Chinese Pharmacol Bull. (2009) 25:270–4.

