# Supplementary material for: SiNiSan Ameliorates the Depression-Like Behavior of Rats That Experienced Maternal Separation Through 5-HT1A Receptor/CREB/BDNF Pathway
Source: Front Psychiatry. 2019 Mar 28;10:160. doi: 10.3389/fpsyt.2019.00160 (PMC6447714; doi:10.3389/fpsyt.2019.00160)

**Quality control of Sinisan by HLPC**

Representative chromatograms of Sinisan extract at 240 nm and 3D-HPLC pattern of Sinisan extract by HPLC-DAD. Paeoniflorin(14.240min), liquiritin (17.465min), hesperidin (22.497min), ammonium glycyrrhetate (46.271min) were identified by chemical standards respectively represented by line S3, S4, S5, S6.


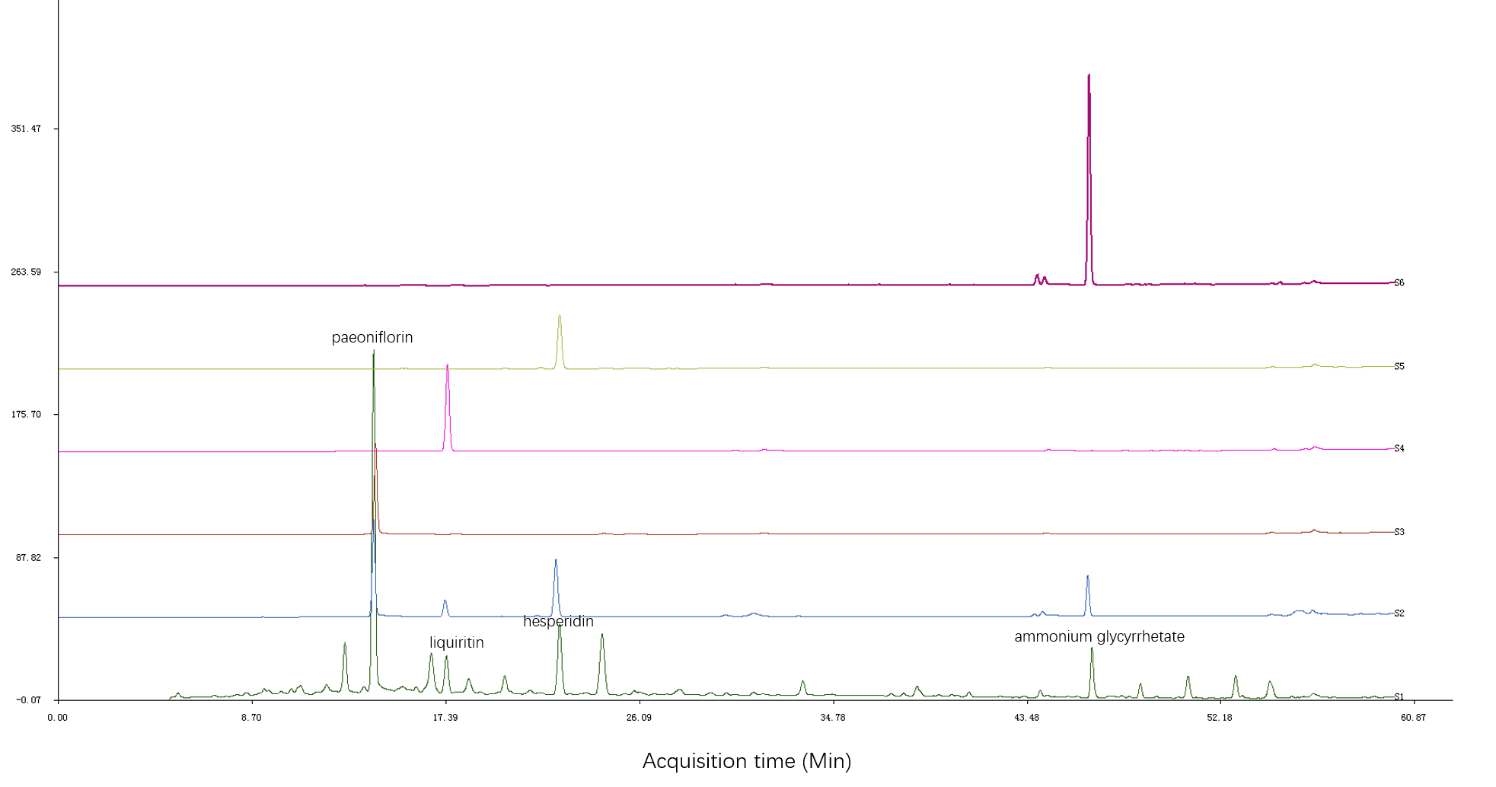

Supplement: Supplementary file 1 [file Data_Sheet_1.docx]
